# Annual Trends in Total Ischemic Time and One-Year Fatalities: The Paradox of STEMI Network Performance Assessment

**DOI:** 10.3390/jcm8010078

**Published:** 2019-01-11

**Authors:** Damian Kawecki, Beata Morawiec, Mariusz Gąsior, Krzysztof Wilczek, Ewa Nowalany-Kozielska, Marek Gierlotka

**Affiliations:** 1 2^nd^ Department of Cardiology, School of Medicine with the Division of Dentistry in Zabrze, Medical University of Silesia, Katowice, Poland, M. Sklodowskiej-Curie Str. 10, 41-800 Zabrze, Poland; beamorawiec@wp.pl (B.M.); ewakozielska@wp.pl (E.N.-K.); 2 3^rd^ Department of Cardiology, School of Medicine with the Division of Dentistry in Zabrze, Medical University of Silesia, Katowice, Silesian Centre for Heart Diseases in Zabrze, Poland, M. Sklodowksiej-Curie Str. 9, 41-800 Zabrze, Poland; m.gasior@op.pl (M.G.); wilky@poczta.onet.pl (K.W.); 3 Department of Cardiology, University Hospital, Faculty of Natural Sciences and Technology, University of Opole, Opole, Poland, Witosa Str. 26, 45-401 Opole, Poland; marek.gierlotka@gmail.com

**Keywords:** total ischemic time, case-fatality, STEMI, network

## Abstract

This study is aimed at assessing trends and relations between total ischemic time, the major quality measure of systemic delay, and case-fatality at the population or patient level in response to growing cardiovascular risk and a constant need to shorten the time to treatment in ST-segment elevation myocardial infarction (STEMI). Data from a prospective nationwide registry of STEMI patients admitted between 2006 and 2013 who were treated with primary percutaneous coronary intervention (PCI) were analyzed. Total ischemic time was calculated as the time from the onset of symptoms to primary PCI and was determined as individual and annual. The primary end-point was one-year, all-cause case-fatality. Among the total 70,093 analyzed patients, temporal trends showed significant decrease in total ischemic time (268 vs. 230 minutes, *p* < 0.001), a worsening of the risk profile and an increase in one-year case-fatality (7.1% vs. 10.8%, *p* < 0.001). In the multivariate analysis, longer individual total ischemic time was a risk factor for higher mortality (HR 1.024, 95%CI 1.015–1.034, *p* < 0.001) and remained significant after adjustment for the year of admission. An inverse relation was observed for the median annual time (HR 0.992, 95%CI 0.989–0.994, *p* < 0.001). Thus, the observed increasing annual trends in case-fatality cannot directly measure the quality of STEMI network performance.

## 1. Introduction

Over the past years, an incremental improvement in the efficiency of treatment in ST-segment elevation myocardial infarction (STEMI) has been achieved worldwide. The percentage of primary percutaneous coronary intervention (PCI) procedures in patients admitted with STEMI increased in Poland from 46% in 2006 to 79% in 2013, and the global one-year case-fatality in the STEMI population has significantly decreased over the years [1,2]. Annually reported early or long-term case fatality rates are still used and considered one of the major measures in the assessment of the quality of care in STEMI. However, considering the constantly growing risk profile of populations, there is a need for careful evaluation and interpretation of annual fatality trends.

The second important discriminant of the quality of care in acute settings is the time to initiation of the specific treatment. According to a thesis originating from animal studies, total ischemic time (TIT), defined as the time from symptom onset to reperfusion, is of major concern in STEMI [3]. Therefore, strong efforts should be put into its maximal shortening. Optimal time frames are well established, addressed and redefined in the current European Society of Cardiology (ESC) guidelines for the management of acute myocardial infarction in patients presenting with ST-segment elevation [4]. However, the available reported delays are still too long [5], even in Poland, a country with one of the highest densities of primary PCI-capable centers and rates of primary PCI procedures for STEMI in Europe [6]. The aim of the study was, first, to assess the trends and relation of total ischemic time and one-year case-fatality during a seven-year period in STEMI patients treated with primary PCI.

## 2. Experimental Section

### 2.1. PL-ACS Registry

The Polish Registry of Acute Coronary Syndromes (PL-ACS) is a nationwide, prospective, ongoing registry of consecutive patients with acute coronary syndrome (ACS) hospitalized in Poland. A detailed protocol and methodology have been presented previously [7]. In brief, the recruitment started from June 2005 after a pilot phase and the final adaptation to cardiology audit and registration data standards (CARDS) [8]. Initially, 535 centers were planning to participate. The selection of centers was reserved for those with at least one of the following departments: coronary care/intensive care unit, cardiology, cardiac surgery, internal medicine, or admitting at least 10 ACS per year. Finally, 417 institutions entered the registry. The inclusion criteria were the diagnosis of unstable angina, non-ST-segment elevation myocardial infarction (NSTEMI) or STEMI, according to previously-described definitions [7].

### 2.2. Current Analysis

The focus of the current analysis were STEMI patients admitted to a primary PCI center not later than 12 hours from the onset of symptoms and treated with primary PCI between 1 January 2006 and 31 December 2013. The exclusion criteria were pre-treatment with fibrinolytic therapy, missing or uncertain data on the time of the onset of symptoms, time of arrival to the primary PCI center, or time of first balloon inflation.

The baseline characteristics, clinical status, left ventricular ejection fraction (LVEF), in-hospital and one-year case fatality were analyzed as trends across the years.

The following time points were reported to the registry: Onset of symptoms and arrival to primary PCI center, both recorded at admission to the primary PCI center; and arterial puncture for coronary angiogram and balloon inflation for PCI, both reported by the interventional team. Total ischemic time was defined as the time from the onset of symptoms to balloon inflation for PCI. For a secondary analysis, the following time intervals were defined and calculated: Symptom-to-door (from the onset of symptoms to the arrival at the primary PCI center) and door-to-balloon (from arrival at the primary PCI center to balloon inflation). In order to check for the accuracy of the total ischemic time and fatality rates as systemic quality measures, the annual and patient-specific total ischemic times were determined. The annual time was defined as the median time for patients within a particular study period and was assigned to all patients admitted in their respective year. The patient-specific time was defined as the time specific to each individual patient included in the study.

### 2.3. Follow-Up

One-year, all-cause case fatality was considered the endpoint for the current analysis. Data on the occurrence of death with the exact date were retrieved from the central healthcare system database and were available for all included patients within one year following STEMI admission.

### 2.4. Statistics

The baseline characteristics, risk factors and hemodynamic characteristics were presented as trends in time per year of admission. Case-fatality trends were presented analogically. The Jonckheere–Terpstra test for continuous variables and the Cochran–Armitage chi^2^ test for categorical variables were used for significance analysis of the trends.

Patient-specific time was divided into 12 categories with 60 min intervals. Annual and patient-specific total ischemic times were plotted against the unadjusted one-year fatality rate for each year to examine group level and individual level relationships. Linear trend lines were fitted to visualize this relation.

The association of total ischemic time with the one-year case fatality was analyzed using Cox proportional hazard regression. Factors included in the analysis were annual and patient-specific total ischemic times, age, sex, hypertension, diabetes mellitus, previous myocardial infarction, previous PCI, anterior STEMI (vs. other electrocardiographic patterns), pre-hospital cardiac arrest, cardiogenic shock at presentation, LVEF and transfer from a regional hospital (vs. direct admission to PCI center). A *p* value of less than 0.05 was considered significant throughout the study. The software used for the analyses were Statistica 12 (StatSoft Inc., Tulsa, OK, USA), IBM SPSS Statistics version 22 (SPSS Inc., Chicago, IL, USA), and GraphPad Prism 6.0 (GraphPad Software, La Jolla, CA, USA).

### 2.5. Role of the Funding Source

There are no founding sources to disclose.

The corresponding author confirms having full access to all the data in the study and having the final responsibility for the decision to submit for publication.

## 3. Results

**Figure 1:**
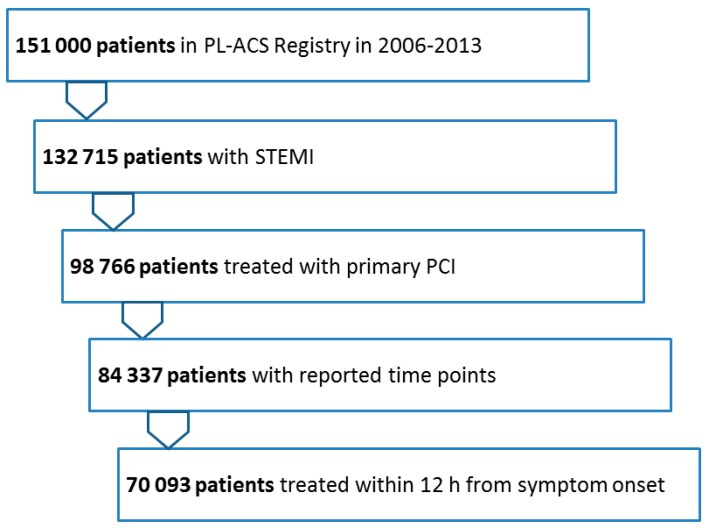
Patient flow. STEMI—ST-segment elevation myocardial infarction, PCI—percutaneous coronary intervention.

From the total of 132,715 STEMI patients reported to the registry from 2006 to 2013, 98,766 were treated with primary PCI. After the exclusion of patients with missing or uncertain time-point data, 84,337 patients remained. Of these 70,093 (83%) were admitted to the PCI center within 12 h of the onset of symptoms (Figure 1).

Temporal trends in baseline characteristics, risk factors, and hemodynamic parameters are presented in Table 1. The analysis showed a significant increase in the rate of hypertension, diabetes mellitus, smoking and obesity, accompanied with older age and a growing rate of females across years. During the analyzed period, there was a systematically rising number of patients with prior PCI, lower LVEF, while the rates of prior CABG and pre-hospital cardiac arrest decreased with time. Rates of previous AMI, diagnosis of anterior STEMI and cardiogenic shock remained relatively unchanged throughout the study period.

**Figure 2:**
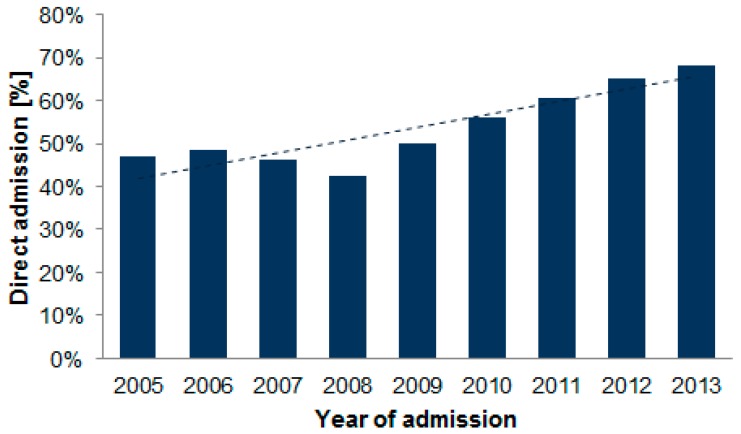
Rate of direct admissions to PCI centers in each year of study period. The fitted linear regression line indicates the trend (*p* < 0.001).

The rates of patients admitted directly to a primary PCI center increased significantly, from 49% in 2006 to 68% in 2013 (*p* < 0.001, Figure 2).

**Figure 3:**
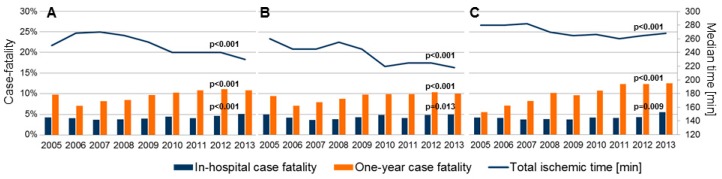
Temporal trends in the median annual total ischemic time matched with rates of in-hospital and one-year mortality 2006–2013 in all patients (**A**), patients admitted directly (**B**), and transferred patients (**C**); *p* value for trend.

The temporal trends for defined time delays showed a statistically significant decrease over the years. The median total ischemic time decreased from the median of 268 minutes in 2006 to 230 minutes in 2013 (15% decrease, *p* < 0.001). Accordingly, the median symptom-to-door time decreased from 195 to 176 minutes (10%, *p* < 0.001) and the median door-to-balloon time decreased from 46 to 42 minutes (9%, *p* < 0.001). Detailed results are presented in Table 2. The temporal trends for particular time spans in the general cohort, for patients admitted directly and for transferred patients are shown in Figure 3, together with case fatality trends. All-cause, in-hospital case fatality in the analyzed period were 4.3%, 5.9% at 30 days, 8.3% at 6 months and 9.8% at 1 year. The in-hospital and one-year case fatality increased significantly over time (Figure 3 and Table 2).

**Figure 4:**
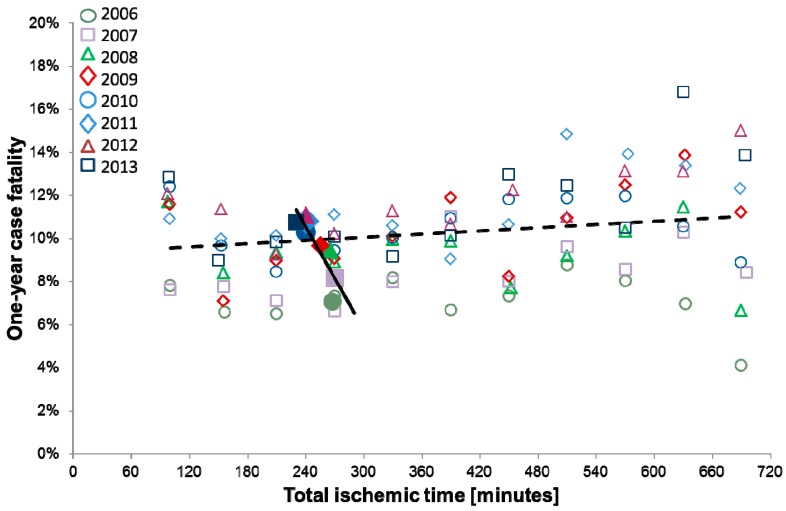
Relation between one-year case fatality and total ischemic time. Open boxes—relation between one-year case fatality and patient-specific total ischemic time within years; Solid boxes—relation between one-year case fatality and annual total ischemic time across years. Linear trend lines represent visual relation; dashed line for patient-specific trend and continuous line for annual trend.

In the univariate Cox regression analysis, the longer total ischemic time was independently associated with an increased case fatality rate at the patient level (HR 1.014 per one hour longer time, 95%CI 1.007–1.022, *p* < 0.001). There was an inverse relation at the annual level reflected in a paradoxically lower risk of fatality with a longer annual total ischemic time (HR 0.911, 95%CI 0.989–0.993, *p* < 0.001) (Table 3). After adjustment for cardiovascular risk factors in a multivariate model, the significance and direction of association of patient-specific and annual total ischemic times with case fatality remained unchanged (HR 1.024, 95%CI 1.015–1.034, *p* < 0.001 and HR 0.992, 95%CI 0.989–0.994, *p* < 0.001, respectively). Longer patient-specific total ischemic time remained a significant risk factor for death, even after adjustment for the year of inclusion (HR 1.024, 95%CI 1.015–1.034, *p* < 0.001). (Table 3). A summary of the associations between patient-specific and annual total ischemic times with fatality rates settled across study period is presented in Figure 4.

## 4. Discussion

The continuous improvement of the efficiency of treatment in STEMI is the constant focus of cardiovascular research. From several factors responsible for the quality of care, annual trends in case fatality and time delays to treatment are considered major discriminants and complemental [9]. However, with regard to the constantly changing risk profile of the general population, annually reported fatality rates should be interpreted carefully. The present study was aimed at reassessing the utility of this measure for the assessment of quality of care in STEMI treatment with primary PCI in the context of temporal trends in delay to treatment.

The major finding of the study was a significant decrease in total ischemic time but a discordant increasing trend in annual case fatality throughout the studied period. This paradox confuses previous assumptions of equality in the interpretation of both factors as system quality measures. Further analysis conducted in order to explain this finding confirmed the paradoxical association of shorter total ischemic time with a higher fatality rate when considered annually, even after adjustments. On the contrary, the reduction of the total ischemic time resulted in lower case fatality at the individual patient level. In other words, annual trends in the fatality of STEMI patients treated by primary PCI are not an adequate system quality measure.

Increases in the fatality rate in short-term and one-year follow-up despite a major shortening in total ischemic time merit discussion. This might be due to the increased aging of the population that is associated with an increasing prevalence of co-morbidities and exposure to worse clinical presentation. The thesis is supported by previous reports [10,11]. The advanced aging of the population could also be directly responsible for rising mortality among STEMI patients, as has been discussed previously [12]. We noted important rising trends in the incidence of major cardiovascular risk factors with a worsening of clinical status of admitted patients. This was reflected in higher case fatality for the group over the analyzed seven-year period. Nevertheless, reducing delays to treatment improved survival in each individual patient, independently of their risk profile.

The inconsistent character of the total ischemic time drives consideration of particular components as the target for the reduction of delays. Based on similar findings for the patient-specific and annual influence on door-to-balloon time published previously, we postulate that the decreasing trend in total ischemic time was mainly driven by reduction in pre-primary PCI center delay [13]. In our analysis, door-to-balloon time was relatively stable across the years of analysis, thus patient-related and systemic but pre-PCI center delays should be the focus of analysis to identify further possibilities of reducing ischemic time. It seems that timing plays a role in predicting myocardial salvage and prognosis after reperfusion therapy, which differs in regard to symptom/electrocardiographic-based estimation of delays [14].

The reduction of delays in the delivery of care was substantially influenced by a rising proportion of patients being admitted directly to a primary PCI facility over the years of analysis. This was the effect of a previously described trend towards an increase in percentage of primary PCI procedures in patients admitted with STEMI in Poland, from 46% in 2006 to 79% in 2013. As reported by our group previously, transfer from non-PCI capable centers was an independent risk factor of one-year case fatality in multivariate analysis, independent of total ischemic time [15]. It is important to note that the total ischemic times well beyond the 120 min threshold are crucial for infarct salvage. Ischemic times in the 230–250 min range, reported in our analysis, have identical mortality rates to those shown previously and similar infarct salvage rates as assessed with cardiac magnetic resonance [16]. Although mortality seems to be lower in patients presenting early after the onset of symptoms independent of treatment strategy [17], it has also been proved that there is no benefit in terms of mortality from the optimization of reperfusion strategies in cases with comparable total ischemic times [16].

Considering primary PCI as the method of choice in the treatment of STEMI, the maximal shortening of the total ischemic time should always be considered as the major target in the strategy of STEMI treatment [18]. This is particularly important in high-risk patients and in early presenters, as there is a loss of mortality benefits per each unit of PCI-related time delay in these patients [19]. It would also be of high importance to assess the efficiency of pre-PCI center management in regard to emergency medical service (EMS) transport versus self-presentation to the PCI center over the years. Patients who call EMS for transport to a STEMI center might have shorter total ischemic time mainly because their symptoms are more severe and they call EMS earlier in their course of care. Second, the cath lab is activated as soon as possible after ECG transmission and the decision of admission, which decreases in-hospital delays. Whether this affects case fatality rates remains to be determined.

Another aspect which should be discussed is the modification of outcomes with pre-treatment with fibrinolysis compared to primary PCI for STEMI. The rapid initiation of fibrinolytic therapy can shorten the time to reperfusion and even the mechanical therapy of the culprit lesion. It was shown that the PCI strategy facilitated with fibrinolysis reduced the mortality among STEMI patients, without increasing the risk of stroke or bleeding, compared to primary PCI [20]. It was also postulated that delays in the ischemic time and mechanical reperfusion therapy during STEMI is associated with greater injury to the microcirculation [21] and loss of benefit from manual thrombus aspiration [22]. Further studies on the use of fibrinolysis before hospital admission for STEMI should provide information on possible initial infarct-related artery patency and the likelihood of shock at presentation.

We would like to prevent the misinterpretation of our results and indicate that the lack of benefit in terms of case fatality from the reduction of total ischemic time after PCI treatment at the population level does not affect the interaction at the individual level, where the patient-specific total ischemic time decreased case fatality. Such an outcome reflects micro-level and macro-level associations, terms originating from ecological studies [23]. By analyzing both types of associations, our results allow interference with them and indicate that the total ischemic time was a risk factor for fatality at the individual level alone and remained significant after accounting for other cardiovascular risk factors, clinical presentation and the year of admission. In the era of personalized medicine, it is of utmost importance to find patient-focused best management [24]. Thus, our findings warrant detailed individual consideration of preventive strategies in order to minimize the risk of fatal outcomes if the delivery of care is anticipated to be prolonged.

## 5. Limitations

The study results should be interpreted with the following limitations. First, the correlation of shorter total ischemic time with lower case fatality at the individual level supports a common hypothesis that prognosis in patients with STEMI depends on the time of ischemia. However, the study cannot prove the inference of key components of the total ischemic time with case fatality (time from the onset of symptoms to first medical contact—patient delay—and the efficacy of emergency medical services—system delay), as these data were unavailable for the analysis. Considering relatively stable door-to-balloon time over the study period, further analyses are needed to assess this relation. Second, the conclusions drawn from the study are supported by data only until 2013 and might have evolved accordingly with continuous growth in the use of primary PCI and constant recognition of the importance and improvement in systemic delays.

## 6. Conclusions

In conclusion, despite the increasing annual trend in one-year case fatality, a shorter total ischemic time was associated with a lower risk of death in every year of the study period in patients treated by primary PCI for STEMI. Thus, observed trends in fatality cannot directly measure the quality of STEMI network performance.

## Figures and Tables

**Table 1:** Temporal trends in risk factors, clinical history and clinical presentation 2006–2013.

Variable	Total *n* = 70,093	2006 *n* = 7940	2007 *n* = 7428	2008 *n* = 6618	2009 *n* = 7932	2010 *n* = 10,463	2011 *n* = 9748	2012 *n* = 10,450	2013 *n* = 9514	*p* Value for Trend
**Age**	63 (55–73)	61 (53–71)	62 (54–72)	62 (55–72)	62 (55–72)	62 (55–73)	63 (55–74)	63 (56–74)	64 (56–74)	<0.001
**Age > 75 y**	14,746, 21%	1318, 16.6%	1352, 18.2%	1323, 20%	1539, 20.1%	2268, 21.7%	2248, 23.1%	2407, 23%	2237, 23%	<0.001
**Male sex**	48,248, 68.8%	5569, 70.1%	5226, 70.4%	4532, 68.5%	5540, 69.8%	7333, 70.1%	6630, 68.0%	7167, 68.6%	6251, 65.7%	<0.001
**Hyper-tension**	44,899, 64.1%	4719, 59.4%	4474, 60.2%	4061, 61.4%	5034, 63.5%	6871, 65.7%	6516, 66.8%	6891, 65.9%	6333, 66.6%	<0.001
**Diabetes Mellitus**	15,558, 22.2%	1328, 16.7%	1408, 19.0%	1525, 23.0%	1820, 22.9%	2515, 24.0%	2296, 23.6%	2426, 23.2%	2240, 23.5%	<0.001
**Smoking**	41,609, 59.4%	3481, 43.8%	3565, 48.0%	3930, 59.4%	4914, 62.0%	6576, 62.9%	6200, 63.6%	6816, 65.2%	6127, 64.4%	<0.001
**Obesity**	12,375, 17.7%	1205, 15.2%	1156, 15.6%	1133, 17.1%	1491, 18.8%	1880, 18.0%	1779, 18.2%	1908, 18.3%	1823, 19.2%	<0.001
**Prior AMI**	8166, 11.7%	941, 11.9%	846, 11.4%	701, 10.6%	886, 11.2%	1250, 11.9%	1138, 11.7%	1282, 12.3%	1122, 11.8%	0.063
**Prior PCI**	4889, 7.0%	102, 1.3%	244, 3.3%	396, 6.0%	612, 7.7%	866, 8.3%	812, 8.3%	979, 9.4%	878, 9.2%	<0.001
**PriorCABG**	1429,2.0%	377, 4.7%	285, 3.8%	121, 1.8%	124, 1.6%	136, 1.3%	125, 1.3%	125, 1.2%	136, 1.4%	<0.001
**EF ≤ 35%**	15,200, 27.6%	1519, 27.1%	1376, 26.8%	1314, 27.4%	1678, 26.9%	2308, 28.1%	2161, 26.7%	2477, 28.0%	2376, 28.9%	0.005
**Anterior AMI**	28,240, 40.3%	3219, 40.5%	2973, 40.0%	2631, 39.8%	3175. 40.0%	4233, 40.5%	3916, 40.2%	4241, 40.6%	3852, 40.5%	0.45
**Killip class 3**	1158, 1.7%	118, 1.5%	117, 1.6%	92, 1.4%	116, 1.5%	195, 1.9%	159, 1.6%	197, 1.9%	164, 1.7%	0.013
**Killip class 4**	3315, 4.7%	356, 4.5%	328, 4.4%	274, 4.1%	375, 4.7%	514, 4.9%	438, 4.5%	530, 5.1%	500, 5.3%	<0.001
**Pre-hospital cardiac arrest**	1677, 2.4%	279, 3.5%	262, 3.5%	143, 2.2%	154, 1.9%	242, 2.3%	217, 2.2%	216, 2.1%	164, 1.7%	<0.001

Data presented as median (25th–75th quartile) or *n*, %. AMI—acute myocardial infarction, PCI—percutaneous coronary intervention, CABG—coronary artery bypass grafting, EF—ejection fraction.

**Table 2:** Temporal trends in time intervals and fatalities 2006–2013.

Variable	Total *n* = 70,093	2006 *n* = 7940	2007 *n* = 7428	2008 *n* = 6618	2009 *n* = 7932	2010 *n* = 10,463	2011 *n* = 9748	2012 *n* = 10,450	2013 *n* = 9514	*P* Value for Trend
**Total ischemic time annual [minutes]**	250	268	270	265	255	240	240	240	230	<0.001
	(165–400)	(180–415)	(180–430)	(175–417)	(170–400)	(160–395)	(158–385)	(160–395)	(153–390)	
**Symptom-to-door time [minutes]**	185	195	195	195	195	180	180	180	176	<0.001
	(120–310)	(125–315)	(121–326)	(125–326)	(120–315)	(114–310)	(110–300)	(115–305)	(109–300)	
**Door-to-balloon time [minutes]**	42	46	45	45	41	43	43	44	42	<0.001
	(30–64)	(30–75)	(30–75)	(30–73)	(30–65)	(30–65)	(30–65)	(30–67)	(30–64)	
**In-hospital case fatality**	3007, 4.3%	324, 4.1%	272, 3.7%	252, 3.8%	314, 4.0%	475, 4.5%	402, 4.1%	481, 4.6%	487, 5.1%	<0.001
**One-year case fatality**	6973, 9.8%	561, 7.1%	606, 8.2%	627, 8.5%	767, 9.7%	1077, 10.3%	1053, 0.8%	1158,11.1%	1024,10.8%	<0.001

Data presented as median (25th–75th quartile) or *n*, %.

**Table 3:** Cox proportional regression analysis of one-year case fatality.

	HR	95% CI	*p* Value	HR	95% CI	*p* Value
**Univariate**						
Symptom-to-PCI time patient specific *	1.014	1.007–1.022	<0.001	-	-	-
Symptom-to-PCI time annual ^†^	0.911	0.989–0.993	<0.001	-	-	-
**Multivariable**						
Symptom-to-PCI time patient specific *	1.024	1.015–1.034	<0.001	1.024	1.015–1.034	<0.001
Symptom-to-PCI time annual ^†^	0.992	0.989–0.994	<0.001	1.006	0.998–1.013	0.139
Age >75 years	3.30	3.10–3.52	<0.001	3.30	3.10–3.52	<0.001
Male sex	0.93	0.87–0.99	0.019	0.93	0.87–0.99	0.022
Hypertension	0.87	0.81–0.93	<0.001	0.87	0.81–0.93	<0.001
Diabetes mellitus	1.30	1.21–1.39	<0.001	1.29	1.21–1.38	<0.001
Prior AMI	1.06	0.96–1.17	0.28	1.06	0.96–1.18	0.23
Prior PCI	0.59	0.51–0.69	<0.001	0.59	0.50–0.68	<0.001
Anterior STEMI	1.15	1.08–1.22	<0.001	1.15	1.08–1.22	<0.001
Pre-hospital cardiac arrest	1.82	1.58–2.09	0.001	1.84	1.59–2.11	<0.001
Cardiogenic shock	4.38	4.01–4.80	<0.001	4.39	4.01–4.80	<0.001
Inter-hospital transfer	1.11	1.04–1.18	0.001	1.12	1.05–1.19	0.001
LVEF (per 10% more)	0.62	0.60–0.63	<0.001	0.62	0.60–0.63	<0.001
Year of admission ^†^	-	-	-	1.09	1.05–1.14	<0.001

* per one hour more. ^†^ per one year change. HR—hazard ratio, CI—confidence interval, PCI—percutaneous coronary intervention, AMI—acute myocardial infarction, STEMI—ST-segment elevation myocardial infarction, LVEF—left ventricular ejection fraction.
